# A FAT-PROMOTING BOTANICAL EXTRACT ARTEMISIA SCOPARIA EXERTS GEROPROTECTION IN C. ELEGANS

**DOI:** 10.1093/geroni/igac059.2665

**Published:** 2022-12-20

**Authors:** Bhaswati Ghosh, Hayden Guidry, Maxwell Johnston, Adam Bohnert

**Affiliations:** Louisiana State University, BATON ROUGE, Louisiana, United States; Louisiana State University, Baton Rouge, Louisiana, United States; Louisiana State University, BATON ROUGE, Louisiana, United States; Louisiana State University, BATON ROUGE, Louisiana, United States

## Abstract

Like other biological processes, aging is not random but subject to molecular control. Natural products that modify core metabolic parameters, including fat content, may provide entry points to extend animal lifespan and promote healthy aging. Here, we show that a botanical extract from Artemisia scoparia (SCO), which promotes fat storage and metabolic resiliency in mice, extends the lifespan of the nematode Caenorhabditis elegans by up to 40%. Notably, this lifespan extension depends significantly on SCO’s effects on fat; SCO-treated worms exhibit heightened levels of unsaturated fat, and inhibiting Δ9 desaturases, which oversee biosynthesis of monounsaturated fatty acids, prevents SCO-dependent fat accumulation and lifespan extension. At an upstream signaling level, SCO prompts changes to C. elegans fat regulation by stimulating nuclear translocation of transcription factor DAF-16/FOXO, an event that requires AMP-activated protein kinase under this condition. Importantly, animals treated with SCO are not only long lived but also show improved stress resistance in late adulthood, suggesting that this fat-promoting intervention may enhance some aspects of physiological health in older age. These findings identify SCO as a natural product that can modify fat regulation for longevity benefit and add to growing evidence indicating that elevated fat can be pro-longevity in some circumstances.

